# On-Line Multi-Frequency Electrical Resistance Tomography (*mf*ERT) Device for Crystalline Phase Imaging in High-Temperature Molten Oxide

**DOI:** 10.3390/s22031025

**Published:** 2022-01-28

**Authors:** Prima Asmara Sejati, Noritaka Saito, Yosephus Ardean Kurnianto Prayitno, Koji Tanaka, Panji Nursetia Darma, Miku Arisato, Kunihiko Nakashima, Masahiro Takei

**Affiliations:** 1 Department of Mechanical Engineering, Division of Fundamental Engineering, Graduate School of Engineering, Chiba University, Chiba 263-8522, Japan; prima.asmara.s@ugm.ac.id (P.A.S.); yosephus.ardean@ugm.ac.id (Y.A.K.P.); k_t19971022@chiba-u.jp (K.T.); panji.nursetia@chiba-u.jp (P.N.D.); masa2@chiba-u.jp (M.T.); 2 Department of Electrical Engineering and Informatics, Vocational College, Universitas Gadjah Mada, Yogyakarta 55281, Indonesia; 3 Department of Materials, Kyushu University, Fukuoka 819-0395, Japan; arisato.miku.142@s.kyushu-u.ac.jp (M.A.); nakasima@zaiko.kyushu-u.ac.jp (K.N.); 4 Department of Mechanical Engineering, Vocational College, Universitas Gadjah Mada, Yogyakarta 55281, Indonesia

**Keywords:** multi-frequency electrical resistance tomography, total harmonic distortion, thermal noise, molten oxide, crystalline phase imaging

## Abstract

An on-line multi-frequency electrical resistance tomography (*mf*ERT) device with a melt-resistive sensor and noise reduction hardware has been proposed for crystalline phase imaging in high-temperature molten oxide. The melt-resistive sensor consists of eight electrodes made of platinum-rhodium (Pt-20mass%Rh) alloy covered by non-conductive aluminum oxide (Al_2_O_3_) to prevent an electrical short. The noise reduction hardware has been designed by two approaches: (1) total harmonic distortion (THD) for the robust multiplexer, and (2) a current injection frequency pair: low $f^{L}$ and high $f^{H}$, for thermal noise compensation. THD is determined by a percentage evaluation of *k-*th harmonic distortions of ZnO at f=0.1~10,000 Hz. The $f^{L}$ and $f^{H}$ are determined by the thermal noise behavior estimation at different temperatures. At  f <100 Hz, the THD percentage is relatively high and fluctuates; otherwise, THD dramatically declines, nearly reaching zero. At the determined $f^{L}\geq$ 10,000 Hz and $f^{H}\approx$ 1,000,000 Hz, thermal noise is significantly compensated. The on-line *mf*ERT was tested in the experiments of a non-conductive Al_2_O_3_ rod dipped into conductive molten zinc-borate (60ZnO-40B_2_O_3_) at 1000~1200 °C. As a result, the on-line *mf*ERT is able to reconstruct the Al_2_O_3_ rod inclusion images in the high-temperature fields with low error, $\varsigma_{f^{L},\;T}$ = 5.99%, at 1000 °C, and an average error$\;\langle \varsigma_{f^{L}}\rangle$ = 9.2%.

## 1. Introduction

In steel industries, new functional steel materials are being developed for next-generation car and aerospace materials. One of the critical points of developing new functional steel materials is the crystalline phase behaviors in high-temperature molten oxide, which influences steel material quality [1]. Particularly, the spatio-temporal distribution of the oxide crystal volume ratio, φ, in the crystalline phase behaviors is an advantageous key parameter to observe the crystallinity in a high-temperature molten oxide. To observe the product quality control of new steel materials, a reliable measurement method of the spatio-temporal distribution of oxide crystal φ is highly demanded. Several conventional off-line measurement methods were used to observe the crystalline behaviors, which are scanning electron microscopy (SEM) [2], X-ray diffraction (XRD) [3], differential scanning calorimetry (DSC), and differential thermal analysis (DTA) [4]. These measurement methods are able to observe the crystalline behaviors, but they are limited for observing its morphology and distribution. In order to enhance the measurement quality, thermogravimetric and differential thermal analysis (TG-DTA) was introduced for the recent steel industry application as a high-standard comprehensive measurement method. TG-DTA is mainly able to measure the mass and thermal change in the high-temperature heated materials during the melting, solidification, crystallization hardening, or transition. Still, TG-DTA as an off-line measurement method is not able to achieve the on-line spatio-temporal distribution of oxide crystal φ of the high industrial demands.

In order to achieve the on-line measurement methods in molten oxide, previously, several electrical fundamental phenomena were reported. For instance, the oxide crystallization phase in molten CaO-SiO_2_-TiO_2_ slag shows the dramatic change of electrical conductivity, σ, due to temperature drops [5]. Besides, an electrical permittivity, ε, which is inversely proportional to reactance, also shows a strong relationship to the changes in oxide crystal φ during the temperature drops [6]. Based on this potential, both electrical properties reflecting in the real and imaginary parts of complex resistance are essential for detecting the crystalline phase in molten oxide. However, a conventional electrical resistance measurement does not provide adequate imaging of the spatio-temporal distribution of oxide crystal φ in high-temperature molten oxide.

In order to perform the adequate imaging of the spatio-temporal distribution of oxide crystal φ in high-temperature molten oxide, electrical impedance tomography (EIT) [7] was proposed. Recently, EIT was improved as frequency difference EIT (*fd*EIT) [8], also known as multi-frequency EIT (*mf*EIT) [9], which has high potential as a measurement device for the on-line spatio-temporal distribution. The *mf*EIT can be simplified as multi-frequency electrical resistance tomography (*mf*ERT) by focusing on the real part of the impedance for interpreting the σ distribution of the measured objects for reconstructing the images using a comprehensive image reconstruction algorithm. Although the *mf*ERT application is able to reconstruct the images of the measured objects in various temperature differences, using *mf*ERT it is difficult to achieve the image reconstruction of the spatio-temporal distribution of oxide crystal φ in high temperatures due to the limitation of *mf*ERT, which is designed only for room temperature application. 

In the case of higher room temperature applications, several *mf*ERTs were implemented at intermediate temperatures. For instance, *mf*ERT as a thermal estimator during hyperthermia at 27~41 °C [10] and the plastic inclusion imaging in the plastic extruder at 27~100 °C [11]. Even though *mf*ERT is able to perform in higher room temperature applications, it is difficult to apply *mf*ERT in high-temperature fields of 1000~1200 °C. The challenges for high-temperature field applications are related to the complexity of the sensor’s material selection, especially for selecting the sensor’s material with a sufficient melting point, and the design of noise reduction hardware, which is able to avoid the harmonic distortion due to the non-linearity of high-temperature molten oxide. Therefore, in this paper, we propose a new concept of an on-line multi-frequency electrical resistance tomography (*mf*ERT) device for the high-temperature spatio-temporal distribution of oxide crystal φ with two original aspects, which are: (1) the architecture of the melt-resistive sensor and (2) the design of noise reduction hardware, which consists of the specified total harmonic distortion (THD) for the robust multiplexer, and current injection frequency determination: low $f^{L}\;$ and high $f^{H}$ frequencies, for compensating the thermal noise. Three objectives to clarify the on-line *mf*ERT are presented as: (1) current injection frequencies determined by estimating the thermal noise behavior at different temperatures, (2) experimental evaluation of the on-line *mf*ERT for mimicking the spatio-temporal distribution of oxide crystal φ by an Al_2_O_3_ rod as an inclusion at different temperatures, and (3) accuracy evaluation of the on-line *mf*ERT reconstructed images.

## 2. Melt-Resistive Sensor and Noise Reduction Hardware under High-Temperature Fields

### 2.1. Melt-Resistive Sensor

**Figure 1:**
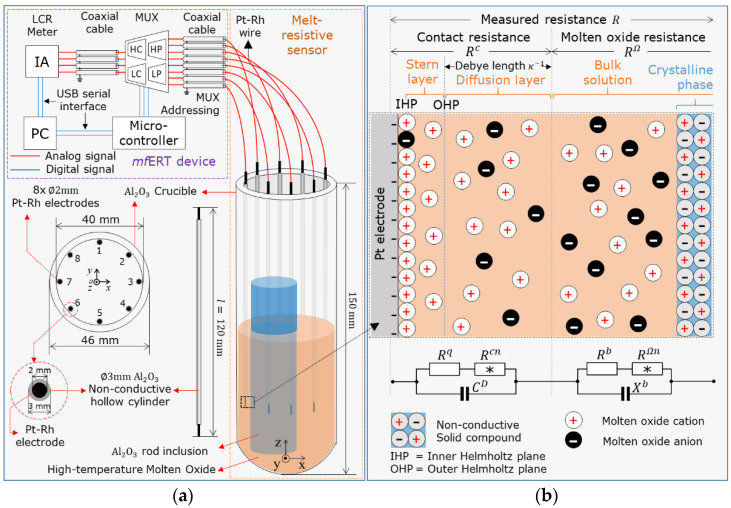
Schematic view of (**a**) the overall on-line *mf*ERT device and (**b**) the resistance measurement of molten oxide.

The first original aspect of the material selection in high-temperature fields is proposed by a melt-resistive platinum-rhodium electrode (Pt-Rh) attached to a crucible. Pt-Rh is a metallic material that possesses great physical properties, such as very high conductivity, $\sigma^{Pt}=$ 1.02 × 10^7^ S/m, and a high melting point at 1773.55 °C [12], which is an ideal material for the melt-resistive electrodes under high-temperature fields in a crucible. In this study, the experimental setup was composed of the melt-resistive sensor attached to the Al_2_O_3_ crucible furnace, as shown in the orange dashed-line box in Figure 1a. The melt-resistive sensor consists of eight cylindrical electrodes made of platinum-rhodium (Pt-20mass%Rh) alloy with a diameter $\emptyset^{\mathrm{Pt}}$ = 2 mm, which is shielded by non-conductive hollow cylinders made of aluminum oxide (Al_2_O_3_) with a diameter $\emptyset_{\;}^{{\mathrm{Al}}_{2}O_{3}\;}$ = 3 mm, which are dipped into the crucible at regular circumference. In order to prevent an electrical short, the Pt-Rh electrode and the Al_2_O_3_ shield are set and maintained with no gap.

### 2.2. Noise Reduction Hardware under High-Temperature Fields

The second original aspect of the resistance measurement under high-temperature fields is the noise reduction hardware. The schematic view of the overall on-line *mf*ERT device under high-temperature fields is shown in Figure 1a. To develop the noise reduction hardware, two strategic concepts are considered, which are: (1) total harmonic distortion (THD) for the robust multiplexer design, and (2) the current injection frequency pair: low $f^{L}$ and high $f^{H}$ frequencies, for compensating the thermal noise.

#### 2.2.1. Total Harmonic Distortion for Robust Multiplexer

**Figure 2:**
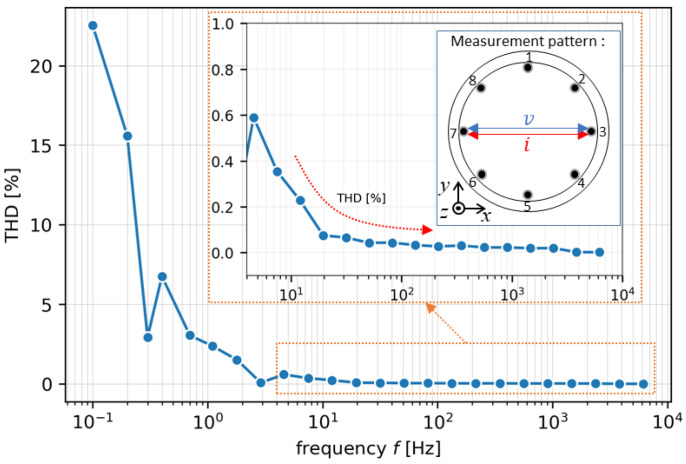
A ten *k-*th harmonic THD plot in the case of ZnO material at I=1 mA under the range of f = 0.1~10,000 Hz.

THD is expressed as the ratio of the signal component to its harmonic signal response caused by the non-linearity of the multiplexer. By applying THD, the aggregated signal-to-noise ratio (SNR) of hardware during non-linear measurement is obtained. Since zinc oxide (ZnO) is the common inorganic compound used as a background in the molten oxide, the electrical properties of ZnO are selected as the assumption under the defined resistivity [13], $R^{\mathrm{ZnO}}$, as shown in Table 1. Here, the THD percentage is estimated by a simulation study. Figure 2 shows a ten *k*-th harmonic THD plot in the case of ZnO material at I=1 mA under the range of  f=0.1~10,000 Hz. Evidently, at the lower frequency  f < 100 Hz, the percentage of THD is relatively high and fluctuates. Otherwise, THD at f ≥ 100 Hz declines dramatically, even nearly reaching zero.

In terms of resistance measurement by current injection as the signal component in THD, the THD represents the residual measured voltage by only removing the fundamental frequency in the frequency domain [14],

(1) $$\mathrm{THD}=\frac{\sqrt{\sum_{k=2}^{+\infty }U_{k}^{2}}}{U_{1}}100\%$$

where $\;U_{1}$ is the root-mean-square (RMS) of the measured fundamental voltage, and $U_{k}$ is the measured RMS voltage at *k-*th harmonic voltage. Using Ohm’s law, the measured resistance is then expressed as $R=F\left|u_{1}\left(t\right)\left|/F\right|i(t)\right|$, where F  is the Fourier transform of the given fundamental voltage signal, $\;u_{1}\left(t\right)$, and current signal,  i(t), in the time domain [15].

As shown in Figure 1b, the measured resistance, R , of the on-line *mf*ERT device consists of the hardware resistance, ${\;R}_{\;}^{h}$**,** and the sensor resistance, $R^{s}\;$, which is defined as ${R=R}_{\;}^{h}{+R}_{\;}^{s}$. Mainly, $R^{h}\;$ consists of coaxial cable resistance and the switching device’s resistance, which contains four multiplexer (MUX) chips to switch the measurement point required by the LCR meter: high current (HC), high potential (HP), low potential (LP), and low current (LC). In the case of ideal hardware, $R^{h}$ is considered to be neglected by proper design due to the high-temperature molten oxide non-linear measurement, and hence, the main component of $R_{\;}\;$ only depends on $\;R^{s}$. We specified the multiplexer of on-line *mf*ERT in a 4 × 8-channel ADG1408 [16] with very low THD, 0.025% (tested at load resistance $R^{L}=$ 110 Ω under 15 Vp-p power supply in the range of f = 20~20,000 Hz). In addition, the SNR of our *mf*ERT device is 55 dB at *f* = 1,000,000 Hz, which meets the standard requirement of an ERT-based device for image reconstruction [17].

#### 2.2.2. Current Injection Frequency Pair: Low $f^{L}$ and High $f^{H}$ Frequencies, to Compensate the Thermal Noise

**Figure 3:**
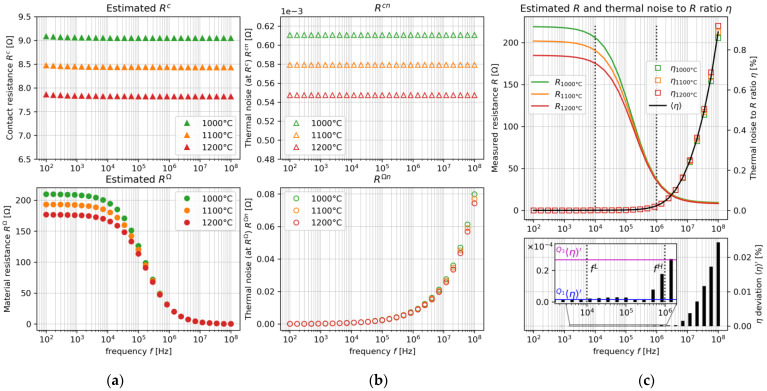
The plots of (**a**) estimated $R^{c}$ and $R^{\Omega }$, (**b**) thermal noise $R^{cn}$ and $R^{\Omega n}$, and (**c**) estimated R and thermal noise to R ratio, η.

To compensate the thermal noise, the relevant frequency pair is determined. Under high-temperature fields, thermal noise known as Johnson–Nyquist noise is unavoidable. Figure 3a–c show the plots of the estimated contact resistance, $R^{c}$, and material resistance, $R^{\Omega }$, thermal noise (at $R^{c}$), $R^{cn}$, and thermal noise (at $R^{\Omega }$), $R^{\Omega n}$, and the estimated R and thermal noise to R ratio, η , under Table 1 assumptions. The measured R  in Figure 3a contains both $R^{cn}\;$ and $R^{\Omega n}$, each of which has the thermal noise component, as shown in Figure 3b. It is shown that the thermal noise is significantly elevated due to the changes of absolute temperature, T, in the low- and high-frequency regions. In contrast, the thermal noise is compensated on certain frequency pairs, as shown in Figure 3c. 

Here, $R^{cn}\;$ and $R^{\Omega n}\;$ depend on T, and are expressed as:

(2) $$R^{cn}=\frac{\sqrt{{4R}^{q}k^{B}{T\Delta f}^{L}}}{I}\;$$

and

(3) $$R^{\Omega n}=\frac{\sqrt{{4R}^{b}k^{B}{T\Delta f}^{H}}}{I}$$

where $R^{q}\;$ is the charge transfer resistance, $R^{b}\;$ is the bulk resistance, $k^{B}\;$ is the Boltzmann’s constant, $\;{\Delta f}^{L}$ is the frequency bandwidth at the lower cut-off frequency, $\;f^{L}$, and $\;{\Delta f}^{H}$ is the frequency bandwidth at the high-frequency limit, $\;f^{H}$. Generally, the frequency bandwidth is defined as ${\Delta f=1/4R}^{\mathrm{sys}}C^{\mathrm{sys}}$ [18], where $R^{\mathrm{sys}}$ is the resistance and $C^{\mathrm{sys}}$ is the capacitance of the system, respectively. In terms of ${\Delta f}^{L}\;$ and ${\Delta f}^{H}\;$ bandwidth in the thermal noise, $f^{L}\;$ is dominated by $\;R^{q}$. At this point, $R^{q}$ declines and the capacitance of the electric double layer (EDL), $C^{D}$, enlarges at the f increment. This phenomenon continues until the resistance of EDL is yielded in a lower value than $\;R^{q}\;$ [19]. At the highest point, $f^{H}\;$ is dominated by $R^{b}$, which is also influenced by bulk reactance at $f^{H}$. Hence, $\;f^{L}$ is expressed as:

(4) $$f^{L}\;\geq \;\frac{1}{{4R}^{q}C^{D}}\;$$

and $f^{H}\;$ is limited at:

(5) $$f^{H}\;\approx \;\frac{1}{{4R}^{b}X^{b}}\;$$

Under the range of the determined $f^{L}$ and $\;f^{H}$, the measured resistance in the on-line *mf*ERT device is evaluated as ${R=R}_{\;}^{c}{+R}_{\;}^{\Omega }$. In the case of current injection through the electrode, $R^{c}$ arises in the EDL and is expressed as a parallel circuit between $R^{q}\;$ and $\;C^{D}$ [20] which is influenced by $R^{\mathrm{cn}}\;$, defined as $R^{c}=\left(R^{q}{+R}^{\mathrm{cn}}\right){||C}^{D}$. As the component of $R^{\mathrm{cn}}$, $R^{q}\;$ is interpreted as:

(6) $$R^{q}=\frac{k^{G}T}{\mathrm{vFI}}\;\;$$

where $\;k^{G}$ is the gas constant, v  is the electron valence, and F is the Faraday constant. Then, $C^{D}$ is established as ions and other charged species from the molten oxide attached to the electrode surface in the stern and the diffusion layers as a dielectric capacitor, $\;C^{D}{=A\varepsilon }_{\;}^{o}\varepsilon^{r}{/k}^{-1}$ [21], where  A  is the surface area of the electrode and $k^{-1}\;$ is the Debye length approximated by [22]:

(7) $$k^{-1}=\sqrt{\frac{\varepsilon^{o}\varepsilon^{r}k^{B}T}{{2F}^{2}𝒾}}\;\;$$

where $\;\varepsilon^{o}\;$ is the vacuum permittivity, $\varepsilon^{r}\;$ is the relative permittivity, and 𝒾 is ionic strength calculated as $𝒾\;=0.5\sum v^{2}c$, with c as the ion concentration of the material. In this case, $\varepsilon^{r}$ is calculated based on $\varepsilon^{r}{=dC}^{b}{/A\varepsilon }^{0}$ [23], where d  is the distance between electrodes and $C^{b}$ is the bulk capacitance of the material [24].

Further, as the next component of  R, $R^{\Omega }\;$consists of $R^{b}$ and ${\;X}^{b}{}_{\;}$and is influenced by $\;R^{\Omega n}$, expressed as $R^{\Omega }=\left(R^{b}{+R}^{\Omega n}\right){||X}^{b}$. Here, $R^{b}\;$ is defined as [25]:

(8) $$R_{\;}^{b}=\frac{d}{\mathrm{AF}\sum z𝓊c}\;\;$$

and $X^{b}$ is expressed as:

(9) $$X^{b}=\frac{1}{{2\pi \mathrm{fC}}^{b}}\;\;$$

where z is the elementary charge, 𝓊 is the mobility constant, and f is the frequency of the exciting alternating current. At different temperatures, $R^{b}$ changes linearly and is expressed by the general equation of $R^{b}{=R}^{b0}\left(1+\mu \Delta T\right)$, where $R^{b0}$ is calculated as $R^{b}$ in Equation (8) at room temperature and μ  is the temperature coefficient of $R^{\mathrm{ZnO}}$.

Based on Equations (2) and (3), one of the thermal noise factors comes from the resistance, which affects the measured resistance,  R. In order to interpret the thermal noise effect on the resistance measurement, the correlation between $R^{cn}{+R}_{\;}^{\Omega n}$ and R is calculated based on the proposed Equations (2)–(9) using Table 1 assumptions. The values above the double lines in Table 1 are the common constants; otherwise, below the double lines are our assumptions. The thermal noise is important to be compensated by determining the proper $f^{L}$ and $\;f^{H}$ in the real R  measurement in the case of unknown $\;R^{q}{,\;C}_{\;}^{D}{,\;R}^{b}{,\;\mathrm{and}\;X}_{\;}^{b}$. The determined ${\;f}_{\;}^{L}$ and $\;f^{H}$ are divided into four sub-steps, which are the calculation of (2-1) the thermal noise to R ratio at the *j*-th temperature sequence, $\eta_{j}$, (2-2) the spatial mean of thermal noise to R ratio 〈η〉, (2-3) deviation of thermal noise to R ratio ${\langle \eta \rangle }^{\prime }$, and (2-4) $\;f^{L}\;$ is determined at the first percentile of ${\langle \eta \rangle }^{\prime }$, 〈η〉 Q1′ and $f^{H}\;$ at the third percentile of ${\langle \eta \rangle }^{\prime }$, 〈η〉 Q3′. In step (2-1), $\eta_{j}$ is calculated by:

(10) $$\eta_{j}=\frac{R_{j}^{\mathrm{cn}}{+R}_{j}^{\Omega n}}{R_{j}}100\%\;\;$$

where j is the temperature sequence defined as 1, 2, …, j, …, J. Then, in step (2-2), the average ratio for all temperatures in each frequency is calculated for 〈η〉, which is expressed as:

(11) $$\langle \eta \rangle =\frac{1}{J}\overset{J}{\underset{j=1}{\sum }}\eta_{j}\;$$

Thus, the resistance distribution among $\eta_{j}$ is obtained by calculating the deviation of the resistance at each different temperature in step (2-3) by:

(12) $$\langle \eta \rangle^{\prime }=\sqrt{\frac{1}{J}\overset{J}{\underset{j=1}{\sum }}{\left(\eta_{j}-\langle \eta \rangle \right)}^{2}}\;$$

Finally, in step (2-4), the determined $f^{L}$ and $f^{H}$ are obtained by analyzing the quartile of the ${\langle \eta \rangle }^{\prime }$. Quartile analysis is adopted as an indicator to determine the threshold for region-merging as it showed less sensitivity to variations of the data distribution [26]. The limit of $\;f^{H}$ frequency is determined at the third quartile of the deviation 〈η〉 Q3′ and the lower cut-off frequency is determined at the first quartile of the deviation 〈η〉 Q1′:

(13) fH ≈ 〈η〉 Q3′

(14) fL ≥ 〈η〉 Q1′

By solving Equations (2)–(14), the thermal noise is compensated at the determined $f^{L}\;\geq$ 10,000 Hz and $\;f^{H}=$1,000,000 Hz, as shown in Figure 3c. Here, under the determined $f^{L}$ and $f^{H}$, R tends to be a non-frequency-dependent region, which is suitable for visualizing the molten oxide under high-temperature fields.

## 3. Experiments

### 3.1. Experimental Setup

**Figure 4:**
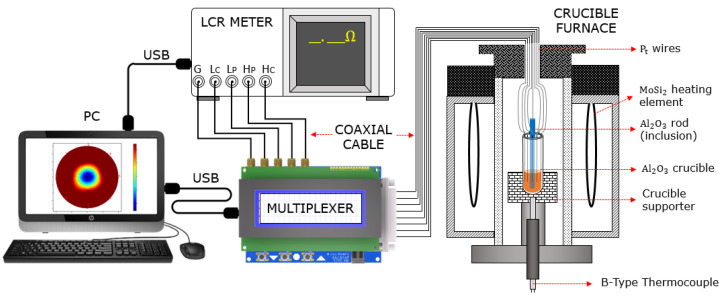
Experimental setup schematic.

Figure 4 shows the experimental setup composed of a crucible furnace, a MUX, an LCR meter, and a personal computer (PC). The Al_2_O_3_ crucible with a melt-resistive sensor was placed inside the crucible furnace held by a crucible supporter. Then, the temperature inside the crucible was controlled by the MoSi_2_ heating element, while the temperature was monitored by a B-type thermocouple. The melt-resistive sensor was connected to an 8-channel MUX with Pt wires which were then spliced to a coaxial cable. The MUX was used as a switching unit to control the melt-resistive sensor electrodes during resistance measurement by the LCR meter. The LCR meter had a resistance measurement accuracy of 0.08% and an excitation frequency range from $f^{\min}$ 4 Hz to $f^{\max}$ 5,000,000 Hz. All measurement data were transmitted to the PC equipped with image reconstruction algorithm software which was adopted from our previous research [27].

### 3.2. Experimental Methods

#### 3.2.1. Measurement Pattern

**Figure 5:**
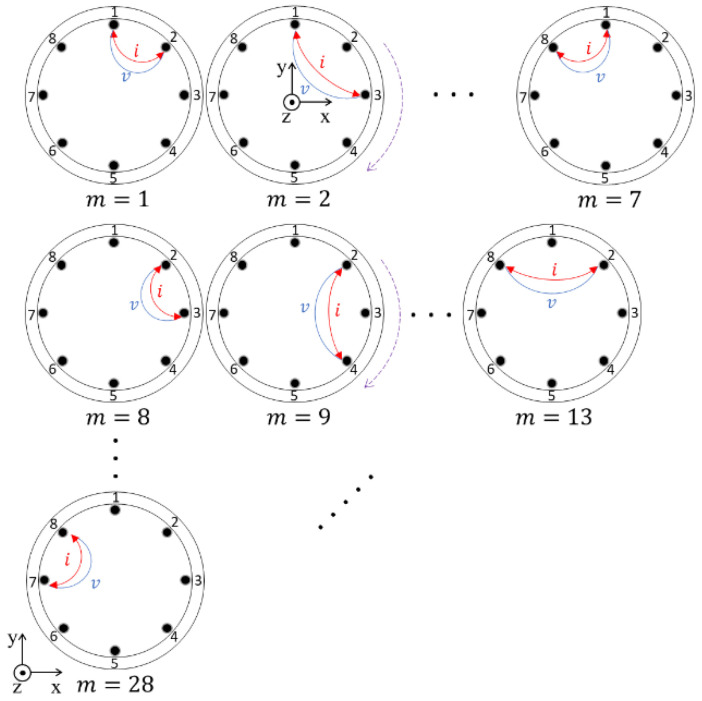
Kelvin-clip measurement patterns.

Figure 5 shows the Kelvin-clip measurement pattern. The measurement pattern conducted in this study was modified from the Quasi adjacent technique [28] by Kelvin-clip as a two-wire measurement mode [29,30]. In this mode, the measurement was conducted by constant current injection and voltage measurements [31]. Then, the number of resistance measurement patterns, M,  was calculated by  E(E−1)/2, where E is the number of the electrodes, and solved as M = 28.

#### 3.2.2. Image Reconstruction

To reconstruct the inclusion image of Al_2_O_3_ among the molten oxide background in high-temperature fields, the conductivity distribution images, σ, were reconstructed based on the standard sensitivity matrix ${\sigma =S}^{T}R$, where $R\;\epsilon \;{ℜ}^{M}\;$ is the M-dimensional measured resistances, R, $\sigma \;\epsilon \;{ℜ}^{N}$ is the N-dimensional vector of conductivity distribution, and $S\;\epsilon \;{ℜ}^{\mathrm{MN}}\;$ is the general sensitivity matrix. σ was represented as ${\left[\sigma_{1}\left(r_{1}\right){,\sigma }_{2}\left(r_{2}\right){,\ldots ,\sigma }_{n}\left(r_{n}\right){,\ldots ,\sigma }_{N}\left(r_{N}\right)\right]}^{T}\;\epsilon \;{ℜ}^{N}$, where $\sigma_{n}\left(r_{n}\right)\;$is the conductivity distribution on the cross-section and $r_{n}{=(x}_{n},y_{n})$ is the Cartesian coordinate of the *n-*th pixel of conductivity distribution. N  is the pixel number in a two-dimensional image. Further, the reconstructed σ from R utilizes the iterative Landweber image reconstruction algorithm (ILBP) [32]:

(15) σ i+1 =σ i  +α(S)T(R−S)σ i )  

where σ i+1  is the reconstructed image at i+1  iteration and α  is the relaxation factor. Here, α  was chosen based on the error reflected in the L-Curve’s elbow [33] by heuristic observation to qualitatively obtain the image reconstruction [34]. The R vector at the determined Δf at every temperature, T, which was defined as ${\Delta R}_{\Delta f,\;T,m}=\left[{\Delta R}_{\Delta f,T,1}{,\ldots ,\;\Delta R}_{\Delta f,T,m}{,\ldots ,\;\Delta R}_{\Delta f,T,M}\right]\;\epsilon \;{ℜ}^{M}$, which was expressed as the resistance difference between one measured resistance, $R_{f_{\;}^{H},T,m}^{\;}\;$, at the determined ${\;f}^{H}\;$ and another measured resistance, $R_{f_{\;}^{L},T,m}^{\;}$, at the determined $f^{L}$ in *mf*ERT. Thus, the determined resistance difference at each m  was calculated based on:

(16) $${\Delta R}_{\Delta f,\;T,m}=\left|\frac{{(R}_{f^{L},T,m}^{\mathrm{obj}\;}{-R}_{f^{L},T,m}^{\mathrm{ref}\;}{)-(R}_{f^{H},T,m}^{\mathrm{obj}\;}{-R}_{f^{H},T,m}^{\mathrm{ref}\;})}{{(R}_{f^{H},T,m}^{\mathrm{obj}\;}{-R}_{f^{H},T,m}^{\mathrm{ref}\;})}\right|\;$$

where ref was the reference condition without the Al_2_O_3_ rod as an inclusion and obj were the Al_2_O_3_ rod inclusions inside the crucible in different conditions.

### 3.3. Experimental Condition

**Figure 6:**
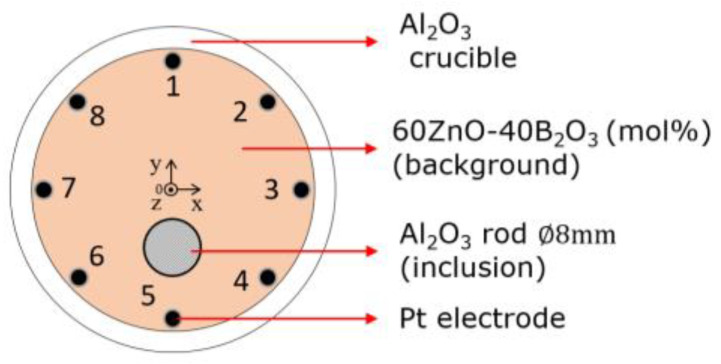
Experimental condition.

Figure 6 shows the experimental condition: the melting background materials inside the Al_2_O_3_ crucible were composed of 60ZnO-40B_2_O_3_ (mol %), which had a liquidus temperature of 988 °C. Meanwhile, the inclusion material was an Al_2_O_3_ rod with $\emptyset^{\mathrm{rod}}\;$ = 8 mm. The crucible temperature was conducted at T=1000, 1050, 1100, and 1200 °C, respectively. The resistance measurement for each electrode pair was conducted for each temperature without inclusions as $R_{\Delta f,\;T}^{\mathrm{ref}}$ and with inclusions as $R_{\Delta f,\;T}^{\mathrm{obj}}$ by the LCR meter’s current injection setting at 1 mA, within the frequency range of 100~5,000,000 Hz.

## 4. Results

### 4.1. Single-Pair Measurement Results at Different Temperatures

**Figure 7:**
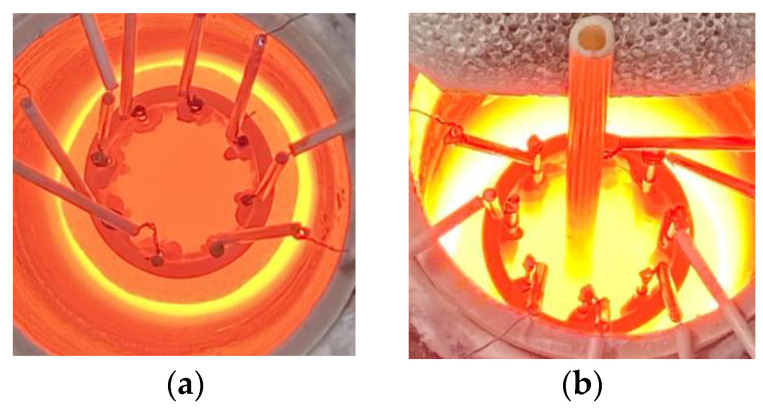
Measurements of the spatial and temporal distribution of oxide crystal φ at 1000 ° C in the crucible furnace mimicked by (**a**) liquid phase of 60ZnO-40B_2_O_3_ as a background and (**b**) with a ∅ 8 mm Al_2_O_3_ rod as an inclusion.

**Figure 8:**
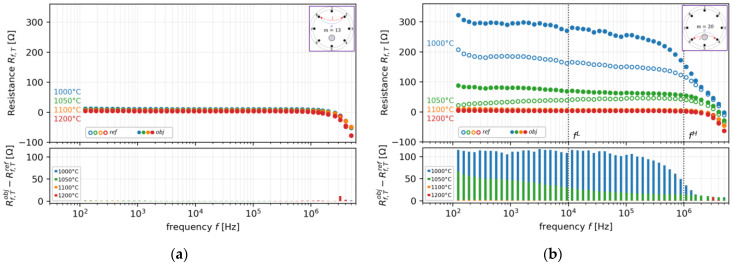
Resistance plot of single-pair measurements at different frequencies and temperatures at (**a**) *m* = 13 and (**b**) at the opposite position, *m* = 20.

Figure 7 shows the measurements of the spatial and temporal distribution of oxide crystal φ at T=1000 °C in the crucible furnace mimicked by (a) liquid phase of 60ZnO-40B_2_O_3_ as a background and (b) with an Al_2_O_3_ rod of $\emptyset^{\mathrm{rod}}\;$ = 8 mm as an inclusion. Then, Figure 8 shows the resistance plot of single-pair measurements at different frequencies and temperatures at (a) *m =* 13 and the opposite position (b) *m* = 20. From this result, it can be seen that our on-line *mf*ERT is capable of measuring the presence of the Al_2_O_3_ rod inclusion at specific electrode pairs. In the case of the absence of the Al_2_O_3_ rod at *m =* 13, as shown in Figure 8a, the resistance difference between the reference and the object, $R_{f,\;T}^{\mathrm{obj}}{\;–\;R}_{f,\;T}^{\mathrm{ref}}$, among all temperatures is very small. In contrast, on the opposite electrode pair at *m =* 20, as shown in Figure 8b, the $R_{f,\;T}^{\mathrm{obj}}{\;–\;R}_{f,\;T}^{\mathrm{ref}}$ value is relatively large.

Moreover, the resistance difference is significantly decreased during the increasing frequency and relaxed at a specific point, as shown in Figure 8b. Hence, it is confirmed that $f^{H}$ is determined at the smallest distribution of resistance difference, which occurred at $f^{H}\;\approx$ 1,000,000 Hz. On the other hand, $f^{L},\;$ which is determined at ≥10,000 Hz, provides the biggest distribution of resistance difference, as shown in Figure 8b. Heuristically, it is shown that the resistance ranges from $f^{L}\;\mathrm{to}\;f^{H}$ are the non-frequency-dependent ranges, which meet the requirement explained in Section 2.

### 4.2. All-Pair Resistance Measurement Results at Different Temperatures

**Figure 9:**
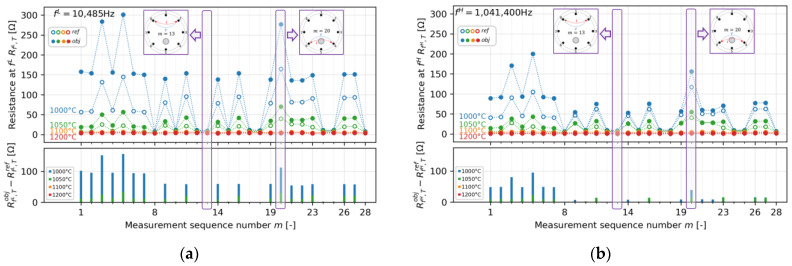

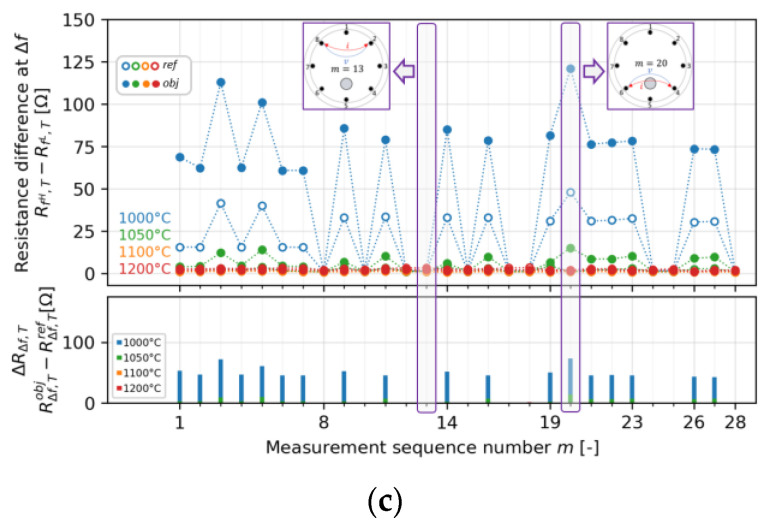
Resistance plots of all measurement pairs in the case of T=1000, 1050, 1100, and 1200 ° C at the determined (**a**) $\;f^{L}$ = 10,485 Hz, (**b**) $f^{H}$ = 1,041,400 Hz, and (**c**) the determined resistance difference, ${\Delta R}_{\Delta f,\;T}$, between the object and the reference.

Figure 9 shows the resistance plots of all measurement pairs in the case of *T* = 1000, 1050, 1100, and 1200 °C at the determined (a) $f^{L}$ = 10,485 Hz, (b) $f^{H}$ = 1,041,400 Hz, and (c) determined resistance difference, ${\Delta R}_{\Delta f,\;T}$, between the object and the reference. As described in Section 4.1, we calculated the ${\Delta R}_{\Delta f,\;T,m}$ distribution of all measurement pairs under the determined $f^{L}f^{H}$ using Equation (16). It is clear that since $\sigma^{{\mathrm{Al}}_{2}O_{3}}{\;<\;\sigma }^{{60\mathrm{ZnO}\text{-}40B}_{2}O_{3}}$, the measurement results clearly show that $R_{\Delta f,\;T,m}^{\mathrm{obj}}{\;>\;R}_{\Delta f,\;T,m}^{\mathrm{ref}}$ at every temperature. On the other hand, the resistance difference level between $R_{\Delta f,\;T,m}^{\mathrm{obj}}\;$ and $R_{\Delta f,\;T,m}^{\mathrm{ref}}\;$ is relatively minor at higher temperatures. Thus, the resistance differences are almost invisible, especially at 1100 and 1200 °C. To perform a better data visualization, the image is reconstructed by Equations (15) and (16) under the determined $f^{L}\;$ and $f^{H}$. 

### 4.3. Image Reconstruction Result

**Figure 10:**
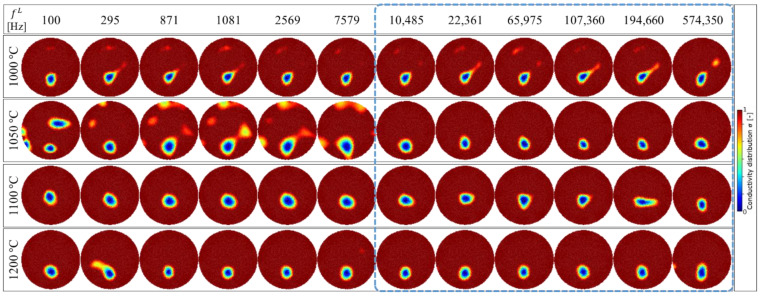
Image reconstruction result of σ in the case of the determined $f^{H}$ = 1,041,400 Hz with several $f^{L}$ frequencies at 1000~1200 ° C. Qualitatively, the satisfactory result occurred at $f^{L}\geq$ 10,485 Hz.

Figure 10 shows the image reconstruction result of σ in the case of the determined $f^{H}$ = 1,041,400 Hz with several $f^{L}$ frequencies at 1000~1200 °C based on Equation (15). The determined $f^{H}\;$ frequency remained fixed at 1,041,400 Hz, with $f^{L}\;$trialled at 100 (minimum), 295, 871, 1081, 2569, 7579, 10,485, 22,361, 65,975, 107,360, 194,660, and 574,350 Hz (maximum), respectively. As shown in Figure 10, the satisfactory result qualitatively occurred at $f^{L}\;\geq$ 10,485 Hz. The noisy result is shown at *T* = 1050 °C at $f^{L}<$ 10,485 Hz. In summary, the determined $f^{L}\geq$ 10,485 Hz (or approximately $f^{L}\geq$ 10,000 Hz) and $f^{H}$ = 1,041,400 Hz (≈1,000,000 Hz) provided the best image reconstruction at all conducted temperatures.

## 5. Discussion

**Figure 11:**
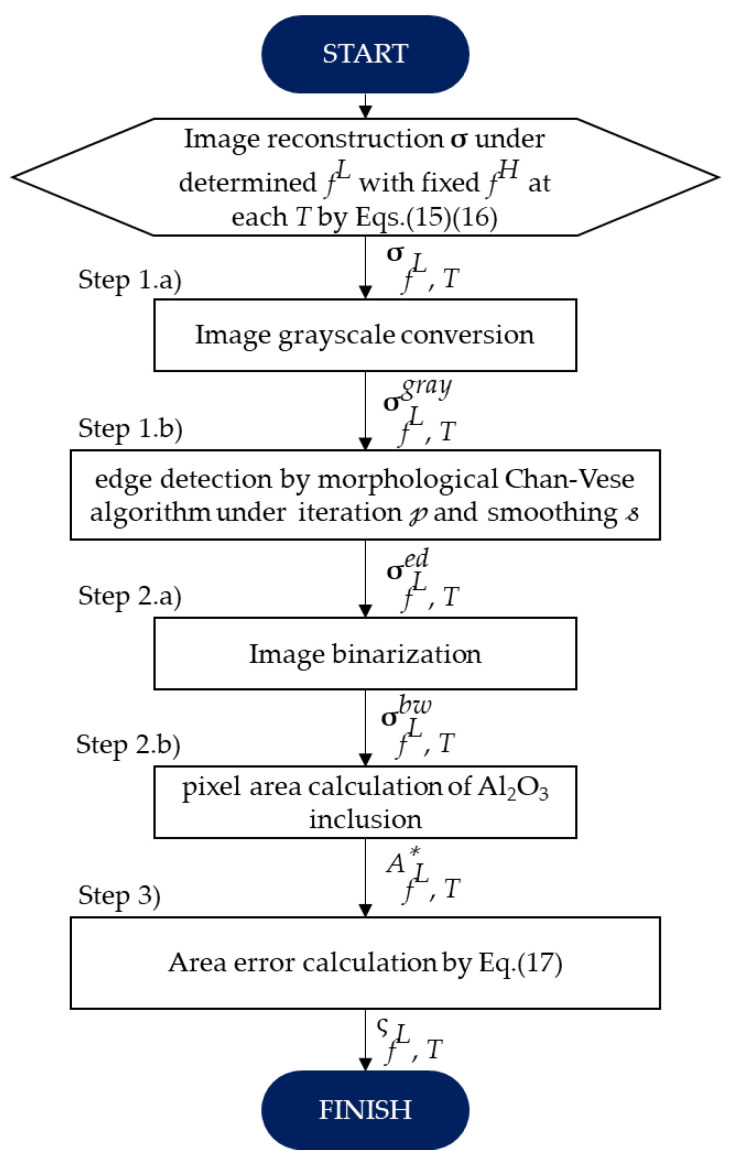
Image reconstruction accuracy evaluation flow chart.

In order to evaluate the image reconstruction accuracy for each temperature, the image reconstruction result shown in Figure 10 is compared with the experiment condition shown in Figure 6 as a true value in three steps, which are: (1) image edge detection by the Chan-Vese segmentation algorithm, (2) image binarization, and (3) area error calculation, as shown in Figure 11.

**Figure 12:**
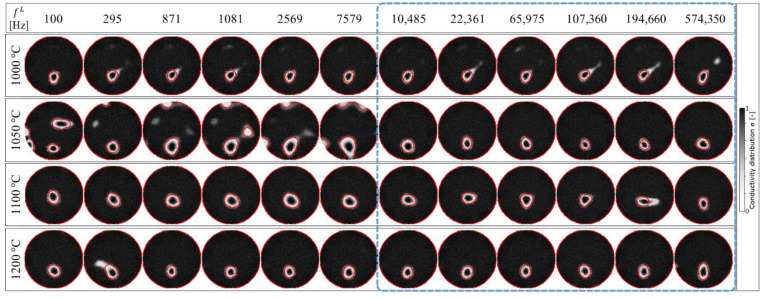
Image edge detection using the Chan-Vese segmentation algorithm.

**Figure 13:**
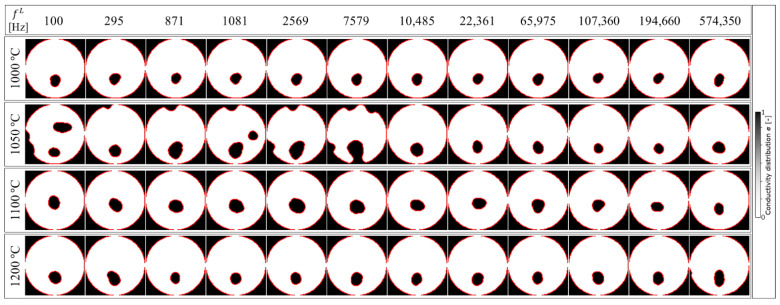
Image binarization.

As the first evaluation in step (1), Figure 12 shows the reconstructed image edge detection using the Chan-Vese segmentation algorithm. This algorithm is based on level sets that are iteratively evolved to minimize the representative image energy. The image energy is defined by weighted values corresponding to the sum of differences intensity from the average value outside, inside, and a term which is dependent on the length of the boundary of the segmented region [35]. Step (1) is divided into two sub-steps, starting with, (1.a) the general image grayscale conversion of σ in Equation (15) under the determined $f^{L}$ and fixed $f^{H}$ at each temperature, $\sigma_{f^{L},\;T}^{\;}$. The gray image output, $\sigma_{f^{L},\;T}^{\mathrm{gray}\;}$, in step (1.a) is then processed in step (1.b), the morphological Chan-Vese segmentation algorithm under iteration, 𝓅, and smoothing parameter, 𝓈. The detected edge, $\sigma_{f^{L},\;T}^{\mathrm{ed}\;}$, in Figure 12 is then binarized as a black and white image, $\sigma_{f^{L},\;T}^{\mathrm{bw}\;}$, in step (2.a), as shown in Figure 13. Further, ${\;\sigma }_{f^{L},\;T}^{\mathrm{bw}\;}$ is computed in step (2.b) to calculate the Al_2_O_3_ inclusion area, $A_{f^{L},\;T}^{*}$. 

**Figure 14:**
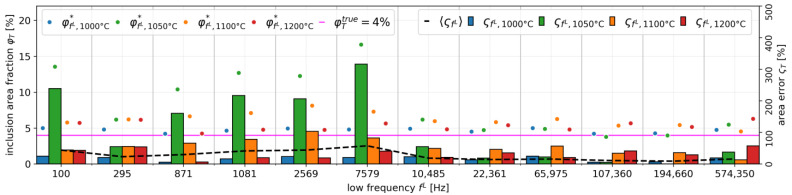
Image reconstruction accuracy result.

Finally, in step (3), the image reconstruction accuracy is defined as area error, $\varsigma_{f^{L},\;T}^{\;}$, at the determined $f^{H}$ = 1,041,400 Hz in several $\;f^{L}$, which is expressed as [36]:

(17) $$\varsigma_{f^{L},\;T}^{\;}=\frac{\left|A_{f^{L},\;T}^{*}{-A}_{f^{L},\;T}^{\mathrm{true}}\right|}{A_{f^{L},\;T}^{\mathrm{true}}}\;\times \;100[\%]\;$$

where $A_{f^{L},\;T}^{\mathrm{true}}$ is the true area of molten zinc borate glass with Al_2_O_3_ inclusion composition and $A_{f^{L},\;T}^{*}$ is the inclusion area of the image reconstruction result. Figure 14 shows the image reconstruction accuracy result as calculated by Equation (17). Here, in order to obtain the best image detection result as shown in Figure 12, we set the iteration 𝓅 = 10 and the smoothing 𝓈 = 9 in step (1.b). Using the aforementioned parameter setting, the $\varsigma_{f^{L},\;1050\;{}^{\circ}C}^{\;}$ result, shown in the green color bar in Figure 14, is relatively higher than $\varsigma_{f^{L},\;1000\;{}^{\circ}C}^{\;}$, $\varsigma_{f^{L},\;1100\;{}^{\circ}C}^{\;}$, and $\varsigma_{f^{L},\;1200\;{}^{\circ}C}^{\;}$. On the other hand, by setting the temperature beyond  𝓈, the $\varsigma_{f^{L},\;T}^{\;}$ at certain temperatures has a possibility to be relatively higher than the other $\varsigma_{f^{L},\;T}^{\;}$. As confirmed in Figure 10, the clearest image reconstruction result occurred at $f^{L}\;\geq$ 10,485 Hz for all temperatures. The best-reconstructed image showed a very low error, $\varsigma_{f^{L},T}$ = 5.99%, under the determined $f^{H}\;\approx$ 1,000,000 and $f^{L}\approx$ 200,000 Hz at 1000 °C, with an average area error $\langle \varsigma_{f^{L}}\rangle$ = 9.2% for all temperatures.

## 6. Conclusions

An on-line multi-frequency electrical resistance tomography (*mf*ERT) device with a melt-resistance sensor and noise reduction hardware has been proposed in a high-temperature crucible for crystalline phase imaging. In this study, we came to several important conclusions, as follows:

By estimating the thermal noise behavior at different temperatures, the best frequency pair was determined at $f^{L}\;\approx$ 10,000 and $f^{H}\;\approx$ 1,000,000 Hz.

The *mf*ERT has the capability to reconstruct the Al_2_O_3_ inclusion images in high-temperature fields ranging from 1000 to 1200 °C.

The accuracy of the image reconstruction had a low error, $\varsigma_{f^{L},T}$ = 5.99%, under the determined $f^{H}\approx$ 1,000,000 and $f^{L}\approx$ 200,000 Hz at 1000 °C, with an average area error $\langle \varsigma_{f^{L}}\rangle$ = 9.2% for all temperatures.

## Figures and Tables

**Table 1:** Assumption values for each variable to estimate R and thermal noise to R ratio,  η, in the case of molten ZnO material at T = 1000~1200 °C.

Symbol	Definition	Value	Unit
$\varepsilon^{0}$	Vacuum permittivity	8.854 × 10^−12^	(F/m)
$k^{B}$	Boltzmann’s constant	1.38 × 10^−23^	(J/K)
$k^{G}$	Gas constant	8.31	(J/(K mol))
z	Elementary charge	1.60 × 10^−19^	(C)
F	Faraday constant	96,500	(J/mol)
$R^{\mathrm{ZnO}}$	Resistivity of ZnO	1	(Ωm)
$C^{b}$	Bulk capacitance of ZnO	5	(nF)
v	Valence of ZnO	14	(-)
μ	Temperature coefficient of $R^{\mathrm{ZnO}}$	0.01	(-)
d	Electrode distance	34	(mm)
r	Electrode radius	1.5	(mm)
I	Current injection amplitude	1	(mA)

## Data Availability

Not applicable.
